# Supplementary material for: Development of anatomically accurate digital organ models for surgical simulation and training
Source: PLoS One. 2025 Apr 9;20(4):e0320816. doi: 10.1371/journal.pone.0320816 (PMC11981654; doi:10.1371/journal.pone.0320816)
Supplement: S1 File — Step by step protocol, also available on protocols.io. (PDF) [file pone.0320816.s001.pdf]

Feb 17, 2025 Version 4

Accurate Digital Organ Models from DICOM V.4

DOI
dx.doi.org/10.17504/protocols.io.bp2l6d19dvqe/v4

Takashi Kimura[1]
[1]Fukushima Medical University

Accurate Digital Organ M…

Takashi Kimura
Fukushima Medical University

OPEN ACCESS

DOI: dx.doi.org/10.17504/protocols.io.bp2l6d19dvqe/v4

Protocol Citation: Takashi Kimura 2025. Accurate Digital Organ Models from DICOM. **protocols.io** **https://dx.doi.org/10.17504/protocols.io.bp2l6d19dvqe/v4**Version created by **Takashi Kimura**

License: This is an open access protocol distributed under the terms of the **Creative Commons Attribution License**, which permits unrestricted use, distribution, and reproduction in any medium, provided the original author and source are credited

Protocol status: Working
We use this protocol and it's working

Created: November 28, 2024

Last Modified: February 17, 2025

Protocol Integer ID: 120059

Funders Acknowledgements:
JSPS KAKENHI
Grant ID: JP19K09100

Disclaimer

This system is provided as an open-source tool for research and educational purposes only. It is not a certified medical device and should not be used for diagnostic, therapeutic, or other medical purposes. The accuracy, completeness, and reliability of the data processed by this system are not guaranteed.
Users are solely responsible for ensuring compliance with applicable laws and regulations in their jurisdiction and for protecting the confidentiality and privacy of any data they use with this system. The developers shall not be held liable for any direct, indirect, incidental, or consequential damages resulting from the use or misuse of this system.
This system is provided "as is," and no warranties, express or implied, are provided. Updates and ongoing support are not guaranteed.
By using this system, you agree to these terms and conditions.

Abstract

This protocol describes the procedure for editing DICOM data to create precise STL-format surface data of organs. Using this method, surface data of organs obtained through auto-segmentation in DICOM viewers such as 3D-Slicer, along with Multi-Planar Reconstruction (MPR) images generated from DICOM data, can be spatially aligned within Blender. By referencing the imaging data, it becomes possible to refine and process the data obtained via auto-segmentation and to create organ data that cannot be captured through auto-segmentation from scratch. As a result, this approach enables the construction of a comprehensive digital dataset of human anatomy that includes all necessary organs.

1

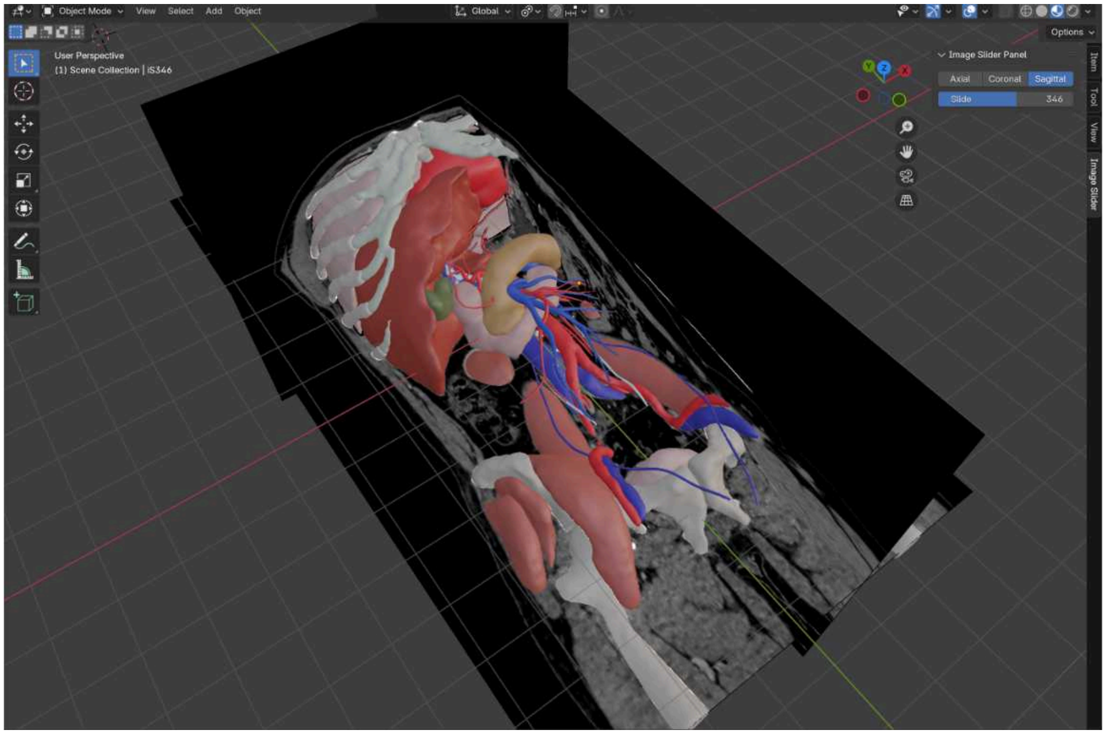

Setup

2 To perform this task, please prepare the following software on your PC.
This document explains the workflow on a Mac (Apple Silicon).

Python: https://www.python.org
(Libraries to be used: pydicom, numpy, matplotlib, sys, glob, bpy)
Blender: https://www.blender.org
3D Slicer: https://www.slicer.org
Horos: https://horosproject.org

JPEG format MPR image creation from DICOM

3

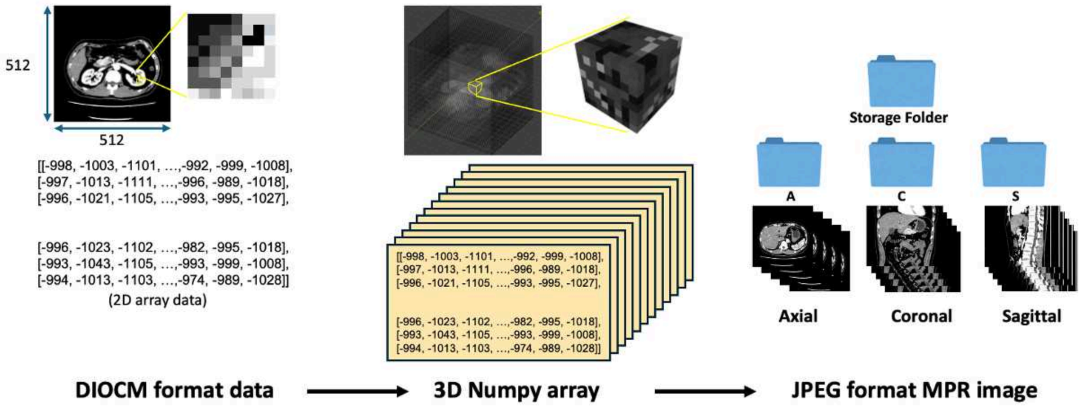

4 Obtain python script from https://github.com/tk1971-Jpn/DICOM-to-JPEG.

5 Run the script on **Jupyter Notebook**.

6 Assign the path of first file in the DICOM data to **path_ct** (enclosed in quotation marks).

7 Create a folder to save the generated JPEG files, and paste the path of this folder into **storage_path**.

8 Retrieve information about the DICOM data each time a cell is executed.

9 Execute the fourth cell and adjust the values of **k** and **l** using the sliders to optimize the image.

10 Input the **l-k** and **l+k** values obtained from the fourth cell into **vmin** and **vmax** in the fifth cell.

11 When you execute the fifth cell, JPEG files will be automatically generated and saved in the specified folder.

Auto-segmentation of organ surface data by 3D-Slicer

12

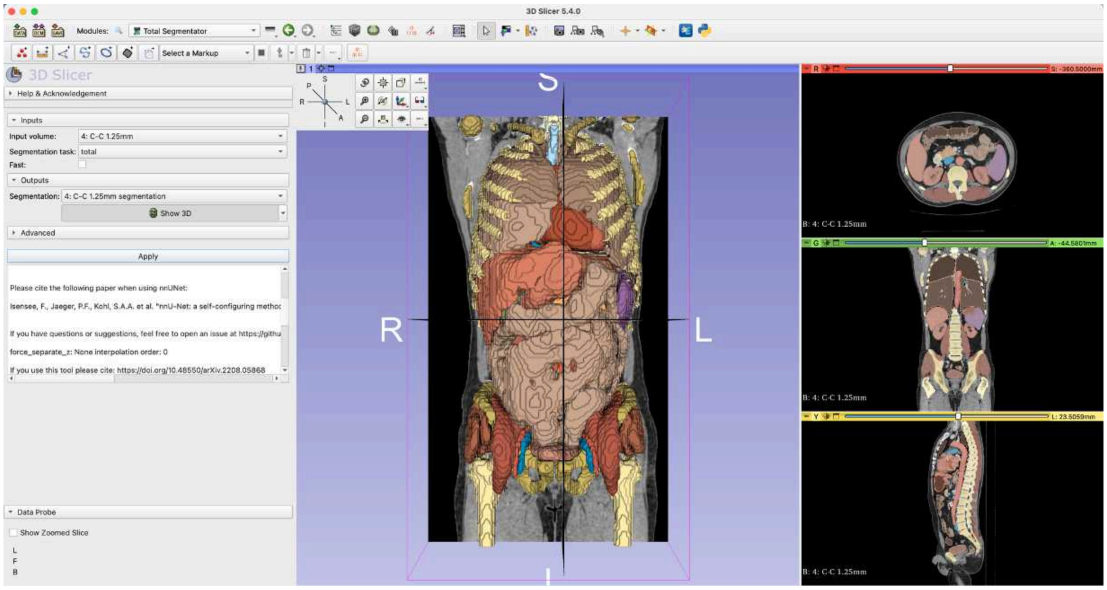

13 Launch **3D-Slicer**.

14 Use the Extension Manager to add **TotalSegmentator**.

15 Import the data you want to convert in **Add DICOM Data** module.

16 Load the required data in **Add DICOM Data** module by selecting and clicking **Load**.

17 Use **TotalSegmentator** to auto-segment organ surface data. After pressing the **Apply** button, you will see two options: "Full resolution (~5 to 50 minutes)" and "Fast (~2 minutes)." Choose the appropriate option based on your needs.

18 Save and extract the organ surface data as an STL file in **Segmentations** module.
- Prepare a folder in advance to save the data.
- Specify the folder in **Destination Folder** under **Export to Files** and press the **Export** button. The data will automatically be saved in the specified folder.

Auto-segmentation of body surface data by Horos (option)

19 In 3D-Slicer, body surface data is not extracted, so the body surface data will be extracted using Horos.

20 Launch **Horos**.

21 Import the data to be used via **Import**.

22 Open the imported data.

23 Select the **3D Surface Rendering** and set the pixel value to -200, then click "OK."

24 Skin, lungs, and other structures will be extracted. Select **Export as STL** from **Export 3D-SR**, specify the file name and save location, and save the file.

Import MPR images into Blender and display them using slider

25

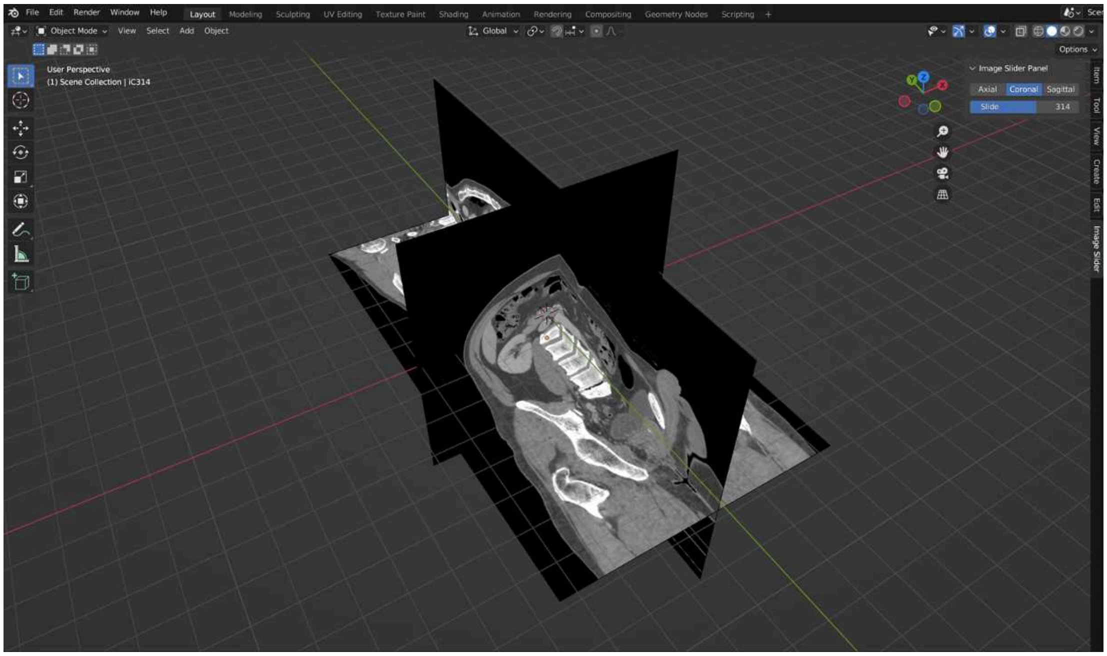

26 Launch **Blender** and select the **Script mode**.

27 Click **New** to prepare for loading a new script.

28 Obtain the python script from https://github.com/tk1971-Jpn/Slider-viewer-in-Blender and paste it into Blender's Script mode.

```
import bpy

pixel_pitch =
ax_slide_number =
size = 0.02
ax_slide_number = 663
ax_slide_distance =
folder_path = '/Users/tkimura/Desktop/TCIA/manifest-
1738120681225/TCGA-BLCA'

cc_scale = ax_slide_number * ax_slide_distance * size
ax_scale = size * pixel_pitch * pixel

if pixel_pitch * pixel < ax_slide_number * ax_slide_distance:
    common_scale = cc_scale
else : common_scale = ax_scale


def load_reference_image(image_path, object_name):
    """
    Load an image as an empty object and assign the specified
name.
    """
    try:
        img = bpy.data.images.load(image_path)
    except Exception as e:
        print(f"Failed to load image: {image_path}, Error: {e}")
        return None

    # Add an empty object
    bpy.ops.object.add(type='EMPTY', location=(0, 0, 0))
    empty_obj = bpy.context.object
    empty_obj.empty_display_type = 'IMAGE'
    empty_obj.data = img
    empty_obj.name = object_name

    return empty_obj

class ImageSliderOperator(bpy.types.Operator):
    bl_idname = "object.image_slider_operator"
    bl_label = "Image Slider Operator"

    def execute(self, context):
        path_CT = folder_path + '/'
```

```
        axis_type = context.scene.image_slider_axis
        slider_value = context.scene.image_slider_property


        if axis_type == 'AX':  # Axial: Slide along the Y-axis
(180-degree rotation along Z-axis)
            ax_slides = ax_slide_number
            image_path = path_CT + 'A/A' + str(slider_value) +
'.jpg'
            ax_distance = ax_slide_distance

            # Remove existing image objects
            for obj in bpy.context.scene.objects:
                if obj.name.startswith("iA"):
                    bpy.data.objects.remove(obj, do_unlink=True)

            # Load the image
            image_obj = load_reference_image(image_path, "iA" +
str(slider_value))
            if not image_obj:
                return {'CANCELLED'}

            # 180-degree rotation along the Z-axis
            image_obj.rotation_euler = (1.5708, 0, 0)

            # Slide along the Y-axis
            image_obj.location = (0, -(ax_distance / 50) *
((ax_slides + 1) / 2 - slider_value), 0)

            # Adjust the size
            image_obj.scale = (ax_scale, ax_scale, ax_scale)

        elif axis_type == 'COR':  # Coronal: Slide along the Z-
axis (180-degree rotation along Y-axis)
            cor_slides = pixel
            image_path = path_CT + 'C/C' + str(slider_value) +
'.jpg'
            size_cor = ax_slide_number * ax_slide_distance * size

            # Remove existing image objects
            for obj in bpy.context.scene.objects:
                if obj.name.startswith("iC"):
                    bpy.data.objects.remove(obj, do_unlink=True)

            # Load the image
```

```
            image_obj = load_reference_image(image_path, "iC" +
str(slider_value))
            if not image_obj:
                return {'CANCELLED'}

            # 180-degree rotation along the Y-axis
            image_obj.rotation_euler = (0, 3.14159, 3.14159)

            # Slide along the Z-axis
            image_obj.location = (0, 0, (pixel_pitch / 50) *
(cor_slides / 2 + 0.5 - slider_value))

            # Adjust the size
            image_obj.scale = (common_scale, common_scale,
common_scale)

        elif axis_type == 'SAG':  # Sagittal: Same as Coronal
(Slide along Z-axis, rotation along Y-axis)
            sag_slides = pixel
            image_path = path_CT + 'S/S' + str(slider_value) +
'.jpg'
            size_sag = ax_slide_number * ax_slide_distance * size

            # Remove existing image objects
            for obj in bpy.context.scene.objects:
                if obj.name.startswith("iS"):
                    bpy.data.objects.remove(obj, do_unlink=True)

            # Load the image
            image_obj = load_reference_image(image_path, "iS" +
str(slider_value))
            if not image_obj:
                return {'CANCELLED'}

            # 180-degree rotation along the Y-axis
            image_obj.rotation_euler = (0, 1.5708, 3.14159)

            # Slide along the Z-axis
            image_obj.location = (-(pixel_pitch / 50) *
(sag_slides / 2 + 0.5 - slider_value), 0, 0)

            # Adjust the size
            image_obj.scale = (common_scale, common_scale,
common_scale)
```

```
        return {'FINISHED'}

class ImageSliderPanel(bpy.types.Panel):
    bl_label = "Image Slider Panel"
    bl_idname = "OBJECT_PT_image_slider"
    bl_space_type = 'VIEW_3D'
    bl_region_type = 'UI'
    bl_category = 'Image Slider'

    def draw(self, context):
        layout = self.layout
        layout.prop(context.scene, 'image_slider_axis',
expand=True)  # Axis selection
        layout.prop(context.scene, 'image_slider_property',
slider=True)  # Slider

def update_axis(self, context):
    """
    Update the slider's maximum value dynamically when the axis is
changed.
    """
    if context.scene.image_slider_axis == 'AX':
        bpy.types.Scene.image_slider_property =
bpy.props.IntProperty(
            name="Slide",
            min=1,
            max=ax_slide_number,
            default=1,
            update=update_slider
        )
    elif context.scene.image_slider_axis in ['COR', 'SAG']:
        bpy.types.Scene.image_slider_property =
bpy.props.IntProperty(
            name="Slide",
            min=1,
            max=pixel,
            default=1,
            update=update_slider
        )

    # Refresh the UI to reflect the changes
    bpy.context.scene.update()

def update_slider(self, context):
    bpy.ops.object.image_slider_operator()
```

```
def register():
    bpy.utils.register_class(ImageSliderOperator)
    bpy.utils.register_class(ImageSliderPanel)
    bpy.types.Scene.image_slider_axis = bpy.props.EnumProperty(
        name="Axis",
        items=[
            ('AX', 'Axial', 'Axial slices'),
            ('COR', 'Coronal', 'Coronal slices'),
            ('SAG', 'Sagittal', 'Sagittal slices')
        ],
        default='AX',
        update=update_axis
    )
    # Initialize the slider property
    bpy.types.Scene.image_slider_property = bpy.props.IntProperty(
        name="Slide",
        min=1,
        max=ax_slide_number,
        default=1,
        update=update_slider
    )

def unregister():
    bpy.utils.unregister_class(ImageSliderOperator)
    bpy.utils.unregister_class(ImageSliderPanel)
    del bpy.types.Scene.image_slider_property
    del bpy.types.Scene.image_slider_axis

if __name__ == "__main__":
    register()
```

29 Assign the data obtained during the execution of **JPEG format MPR image creation from DICOM** to the corresponding variables in the Blender script

29.1 **pixel**(Blender) = pixel(Jupyter)

29.2 **pixel_pitch**(Blender) = pixel pitch(Jupyter)

29.3 **ax_slide_number**(Blender) = number of files(Jupyter)

29.4 **ax_slide_distance**(Blender) = slice distance(Jupyter)

30 Paste the path of the folder* where the JPEG format MPR image data is stored into **path_JPEG**
∗ A folder containing Folder **A**, Folder **C**, and Folder **S**

31 When the script is executed, a tag named **Image Slider** will appear in the sidebar of the Layout mode. You can use the sliders to display any Axial, Coronal, or Sagittal images.

32 *Since the size of the human body far exceeds Blender's workspace, it was scaled down to 1/50 for import.*

Import Organ Surface Data in STL Format into Blender

33

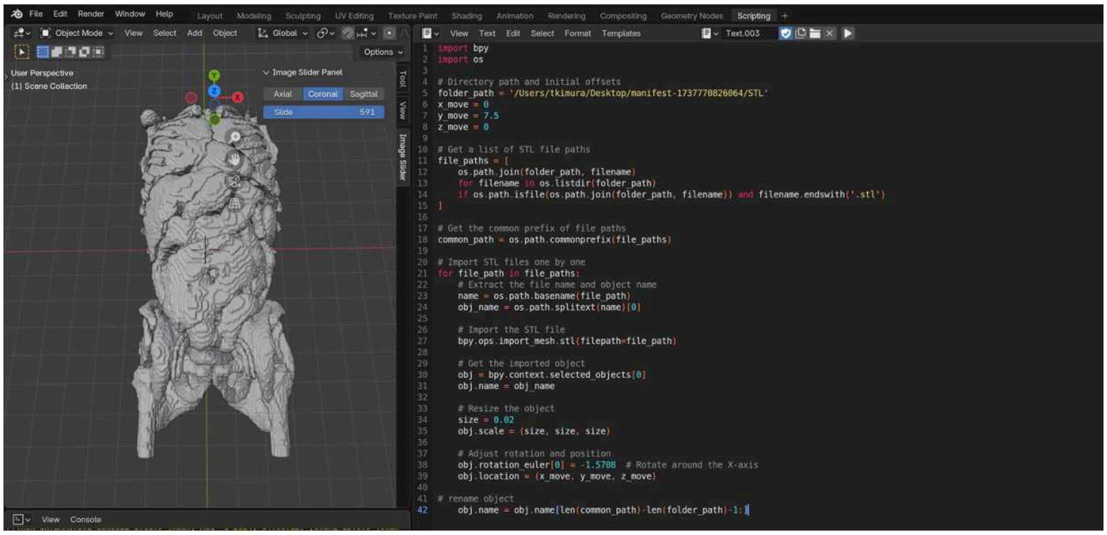

34 Select Script Mode in Blender

35 Click **New** to prepare for loading a new script.

36 Obtain the Python script from https://github.com/tk1971-Jpn/Import-organ-STL-data-into-Blender and paste it into the Blender's Script mode.

```
import bpy
import os

# Directory path and initial offsets
folder_path =
x_move = 0
y_move = 0
z_move = 0

# Get a list of STL file paths
file_paths = [
    os.path.join(folder_path, filename)
    for filename in os.listdir(folder_path)
    if os.path.isfile(os.path.join(folder_path, filename)) and
filename.endswith('.stl')
]

# Get the common prefix of file paths
common_path = os.path.commonprefix(file_paths)

# Import STL files one by one
for file_path in file_paths:
    # Extract the file name and object name
    name = os.path.basename(file_path)
    obj_name = os.path.splitext(name)[0]

    # Import the STL file
    bpy.ops.import_mesh.stl(filepath=file_path)

    # Get the imported object
    obj = bpy.context.selected_objects[0]
    obj.name = obj_name

    # Resize the object
    size = 0.02
    obj.scale = (size, size, size)

    # Adjust rotation and position
    obj.rotation_euler[0] = -1.5708  # Rotate around the X-axis
    obj.location = (x_move, y_move, z_move)

# rename object
    obj.name = obj.name[len(common_path)-len(folder_path)-1:]
```

37 Past the path of the folder where the organ STL data is stored into **folder_path**.

38 When the script is executed, all organ STL data are imported into Blender in bulk, and the object names are appropriately converted during the process.

39 If there is a positional misalignment of the organs (which often occurs), use Blender's "Move" tool to adjust their positions based on the reference image.

40 (Option)Delete all the imported STL data, set the positions, and re-import them. The data used for position adjustments can be obtained from Blender's sidebar under Transform > Location. Based on this data, assign values to the script's **x_move**, **y_move**, and **z_move** (default is 0).
*#Delete all objects: press **A** and then press **X***

41 *Since the size of the human body far exceeds Blender's workspace, it was scaled down to 1/50 for import.*

42 **option:** Option: Use the script below to remesh all objects at once and reduce the file size.

```
import bpy

def remesh_selected_objects(voxel_size=3, remesh_mode='VOXEL'):

    # Get selected mesh objects
    selected_objects = [obj for obj in
bpy.context.selected_objects if obj.type == 'MESH']

    if not selected_objects:
        print("No mesh objects selected.")
        return

    # Apply the remesh modifier to each object
    for obj in selected_objects:
        # Set the active object
        bpy.context.view_layer.objects.active = obj
        bpy.ops.object.modifier_add(type='REMESH')

        # Configure the remesh modifier
        modifier = obj.modifiers[-1]  # Get the latest added
modifier
        modifier.mode = remesh_mode   # Set remesh mode ('VOXEL',
'QUAD', 'SMOOTH', 'SHARP')

        if remesh_mode == 'VOXEL':
            modifier.voxel_size = voxel_size  # Set voxel size

        # Apply the remesh modifier
        bpy.ops.object.modifier_apply(modifier=modifier.name)

    print(f"Remeshed {len(selected_objects)} objects
successfully.")

# Run the script
remesh_selected_objects(voxel_size=3, remesh_mode='VOXEL')
```
