# Supplementary figures and images for: Development of anatomically accurate digital organ models for surgical simulation and training
Source: PLoS One. 2025 Apr 9;20(4):e0320816. doi: 10.1371/journal.pone.0320816 (PMC11981654; doi:10.1371/journal.pone.0320816)

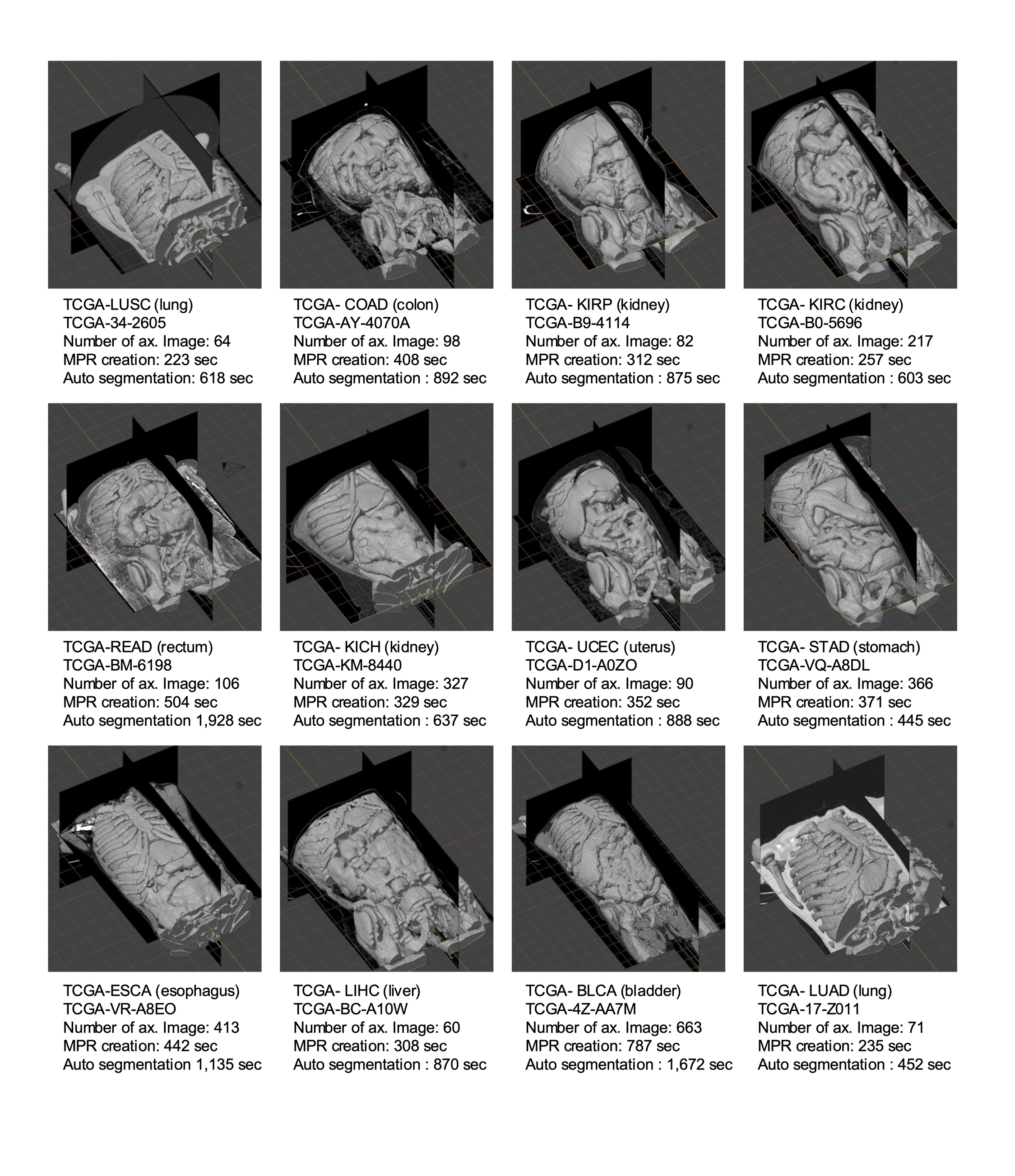

Supplement: S1 Fig — The organ surface data from 12 TCIA cases, displayed in Blender along with the MPR images. (TIF) [file pone.0320816.s003.tif]
