# Supplementary material for: Development of anatomically accurate digital organ models for surgical simulation and training
Source: PLoS One. 2025 Apr 9;20(4):e0320816. doi: 10.1371/journal.pone.0320816 (PMC11981654; doi:10.1371/journal.pone.0320816)
Supplement: Attachment — Submitted filename: Response to Reviewers.docx [file pone.0320816.s005.docx]

**Response to Journal**

We have made the following revisions based on your instructions. Please let us know if any further adjustments are needed.

**Journal Requirements:**

**When submitting your revision, we need you to address these additional requirements.**

**1. Please ensure that your manuscript meets PLOS ONE's style requirements, including those for file naming. The PLOS ONE style templates can be found at** 
[**https://journals.plos.org/plosone/s/file?id=wjVg/PLOSOne_formatting_sample_main_body.pdf**](https://journals.plos.org/plosone/s/file?id=wjVg/PLOSOne_formatting_sample_main_body.pdf) **and** 
[**https://journals.plos.org/plosone/s/file?id=ba62/PLOSOne_formatting_sample_title_authors_affiliations.pdf**](https://journals.plos.org/plosone/s/file?id=ba62/PLOSOne_formatting_sample_title_authors_affiliations.pdf)

**Response to Journal**
I have revised the manuscript according to PLOS ONE's style requirements. Please let me know if any further modifications are necessary.

**2. Please note that PLOS ONE has specific guidelines on code sharing for submissions in which author-generated code underpins the findings in the manuscript. In these cases, we expect all author-generated code to be made available without restrictions upon publication of the work. Please review our guidelines at** [**https://journals.plos.org/plosone/s/materials-and-software-sharing#loc-sharing-code**](https://journals.plos.org/plosone/s/materials-and-software-sharing#loc-sharing-code) **and ensure that your code is shared in a way that follows best practice and facilitates reproducibility and reuse.**

**Response to Journal**
All Python scripts used in the protocol described in this manuscript have been made publicly available in a GitHub repository. A detailed usage guide is provided in the README to ensure ease of use for readers. The main code is also made directly accessible from the protocol registered on protocol.io.

**3. We note you have not yet provided a** [**protocols.io**](http://protocols.io) **PDF version of your protocol and/or a** [**protocols.io**](http://protocols.io) **DOI. When you submit your revision, please provide a PDF version of your protocol as generated by** [**protocols.io**](http://protocols.io) **(the file will have the [protocols.io](http://protocols.io)logo in the upper right corner of the first page) as a Supporting Information file. The filename should be S1_file.pdf, and you should enter “S1 File” into the Description field. Any additional protocols should be numbered S2, S3, and so on. Please also follow the instructions for Supporting Information captions [**[**https://journals.plos.org/plosone/s/supporting-information#loc-captions**](https://journals.plos.org/plosone/s/supporting-information#loc-captions)**]. The title in the caption should read: “Step-by-step protocol, also available on protocols.io.”**

**Please assign your protocol a** [**protocols.io**](http://protocols.io) **DOI, if you have not already done so, and include the following line in the Materials and Methods section of your manuscript: “The protocol described in this peer-reviewed article is published on** [**protocols.io**](http://protocols.io)**(**[**https://dx.doi.org/10.17504/protocols.io.[...]**](https://dx.doi.org/10.17504/protocols.io.%5B...%5D)**) and is included for printing purposes as S1 File.” You should also supply the DOI in the Protocols.io DOI field of the submission form when you submit your revision.**

**If you have not yet uploaded your protocol to** [**protocols.io**](http://protocols.io)**, you are invited to use the platform’s protocol entry service [**[**https://www.protocols.io/we-enter-protocols**](https://www.protocols.io/we-enter-protocols)**] for doing so, at no charge. Through this service, the team at** [**protocols.io**](http://protocols.io) **will enter your protocol for you and format it in a way that takes advantage of the platform’s features. When submitting your protocol to the protocol entry service please include the customer code PLOS2022 in the Note field and indicate that your protocol is associated with a PLOS ONE Lab Protocol Submission. You should also include the title and manuscript number of your PLOS ONE submission.**

**Response to Journal**
We have provided the PDF version of protocols.io as S1_file.pdf. Additionally, we have included the protocols.io DOI in the Material and Method section of the manuscript.
Additionally, information has been added to the Supporting Information at the end of the manuscript.
I have updated the version of the protocol on protocol.io; however, I was unable to add the custom code, manuscript title, or manuscript number during the update.

**4. Thank you for stating the following financial disclosure: 
“TK:
JSPS KAKENHI Grant Number JP19K09100
Japan Society of Promotion of Science(JSPS)**
[**https://www.jsps.go.jp**](https://www.jsps.go.jp)**”**

**Please state what role the funders took in the study. If the funders had no role, please state: "The funders had no role in study design, data collection and analysis, decision to publish, or preparation of the manuscript." 
If this statement is not correct you must amend it as needed. 
Please include this amended Role of Funder statement in your cover letter; we will change the online submission form on your behalf.**

**Response to journal**
I have included the statement, "The funders had no role in study design, data collection and analysis, decision to publish, or preparation of the manuscript." in the cover letter and online submission, as the funders had no involvement. Please update the online submission accordingly if necessary.

**5. Thank you for stating the following in the Acknowledgments Section of your manuscript: 
“This work was supported by JSPS KAKENHI Grant Number JP19K09100”**

**We note that you have provided funding information that is currently declared in your Funding Statement. However, funding information should not appear in the Acknowledgments section or other areas of your manuscript. We will only publish funding information present in the Funding Statement section of the online submission form. 
Please remove any funding-related text from the manuscript and let us know how you would like to update your Funding Statement. Currently, your Funding Statement reads as follows: 
“TK:
JSPS KAKENHI Grant Number JP19K09100
Japan Society of Promotion of Science(JSPS)**
[**https://www.jsps.go.jp**](https://www.jsps.go.jp)**”**

**Please include your amended statements within your cover letter; we will change the online submission form on your behalf.**

**Response to Journal**
As per your instructions, I have removed the funding information from the acknowledgements section.

**6. We note that Figures 3, 4, 5 and 6 in your submission contain copyrighted images. All PLOS content is published under the Creative Commons Attribution License (CC BY 4.0), which means that the manuscript, images, and Supporting Information files will be freely available online, and any third party is permitted to access, download, copy, distribute, and use these materials in any way, even commercially, with proper attribution. For more information, see our copyright guidelines:** [**http://journals.plos.org/plosone/s/licenses-and-copyright**](http://journals.plos.org/plosone/s/licenses-and-copyright)**.**

**We require you to either (1) present written permission from the copyright holder to publish these figures specifically under the CC BY 4.0 license, or (2) remove the figures from your submission:**

**a. You may seek permission from the original copyright holder of Figures 3, 4, 5 and 6 to publish the content specifically under the CC BY 4.0 license.**

**We recommend that you contact the original copyright holder with the Content Permission Form (**[**http://journals.plos.org/plosone/s/file?id=7c09/content-permission-form.pdf**](http://journals.plos.org/plosone/s/file?id=7c09/content-permission-form.pdf)**) and the following text:
“I request permission for the open-access journal PLOS ONE to publish XXX under the Creative Commons Attribution License (CCAL) CC BY 4.0 (**[**http://creativecommons.org/licenses/by/4.0/**](http://creativecommons.org/licenses/by/4.0/)**). Please be aware that this license allows unrestricted use and distribution, even commercially, by third parties. Please reply and provide explicit written permission to publish XXX under a CC BY license and complete the attached form.”**

**Please upload the completed Content Permission Form or other proof of granted permissions as an "Other" file with your submission.**

**In the figure caption of the copyrighted figure, please include the following text: “Reprinted from [ref] under a CC BY license, with permission from [name of publisher], original copyright [original copyright year].”**

**b. If you are unable to obtain permission from the original copyright holder to publish these figures under the CC BY 4.0 license or if the copyright holder’s requirements are incompatible with the CC BY 4.0 license, please either i) remove the figure or ii) supply a replacement figure that complies with the CC BY 4.0 license. Please check copyright information on all replacement figures and update the figure caption with source information. If applicable, please specify in the figure caption text when a figure is similar but not identical to the original image and is therefore for illustrative purposes only.**

**Response to Journal**
Since the copyright holders of Fig3, Fig4, Fig5, Fig6, Fig7, S_Fig1 are the authors themselves, we have created and uploaded an original proof of permission instead of using the recommended Permission Form. If this approach is inappropriate, please provide further instructions.

**7.  Please include captions for your Supporting Information files at the end of your manuscript, and update any in-text citations to match accordingly. Please see our Supporting Information guidelines for more information:** [**http://journals.plos.org/plosone/s/supporting-information**](http://journals.plos.org/plosone/s/supporting-information)**.**

**Response to Journal**
As per your instructions, we have added captions for the Supporting Information files at the end of the manuscript.

**Response to Reviewer #1**

**Reviewer #1: Following a thorough review of the manuscript, the following issues and problems were identified. By addressing these issues, it is anticipated that the paper will exhibit enhanced quality and reliability, thereby more clearly demonstrating the value of the proposed system.**

**Response to Reviewer #1**
We appreciate the review of our manuscript and the valuable comments that have contributed to its improvement.
Based on the feedback received, we revised the content to remove excessive evaluation and reorganized the discussion. The main achievement of this study is the development of a system that imports organ surface data, obtained through deep learning-based automatic extraction techniques such as 3D Slicer, into CAD software. This allows for modifications based on the original image data to create a more ideal representation.
The data generated by this system is expected to be applicable to various surgical simulations, such as surgical planning and training. However, since no evaluation of the simulator has been conducted, we limit our discussion to the potential applications.
Below, we provide our revisions and responses to the comments received.

**1. The methodology is inadequate in terms of detail.- The automatic segmentation process and the deep learning model used are not clearly explained.
In order to enhance reproducibility, it is essential to provide more specific information, including the procedure, the software used, and the parameter settings.**

**Response to Reviewer #1**
We have published the overall workflow of the protocol on protocols.io, while the Python scripts required for its implementation have been uploaded to a GitHub repository making them easily accessible via a link from protocols.io.
Additionally, to enable readers to replicate this protocol using specific TCIA data, we have provided a detailed demonstration on GitHub. This demonstration covers the entire process—from obtaining data from TCIA to setting up the simulation environment in Blender (corresponding to Fig. 3C in the manuscript)—including parameter settings and other necessary configurations.
https://dx.doi.org/10.17504/protocols.io.bp2l6d19dvqe/v4
https://github.com/tk1971-Jpn/Demonstration-of-Organ-data-set-creation

**2. Lack of evaluation metrics- Objective metrics are absent, hindering the evaluation of the effectiveness of the proposed system. For instance, the anatomical accuracy of the generated model and quantitative evaluation of training effects are required.**

**Response to Reviewer #1**
Thank you for pointing out this important aspect. To evaluate the usefulness of this protocol, we obtained data from 12 different projects of The Cancer Genome Atlas (TCGA) within the TCIA dataset, each representing a different type of cancer. We measured the processing time for each step and included the data in S2 File and S1 Fig in the manuscript.
The time required to process the data—from DICOM files to setting up the simulation environment in Blender (corresponding to Fig. 3C in the manuscript)—was approximately 7 minutes on average when using 3D Slicer's Fast mode, which produces a slightly rough surface. When using the Full-resolution mode, which provides more detailed data, the processing time was around 22 minutes. Even when including the time required for launching the program and configuring parameters, the entire process can be completed within 15 to 30 minutes.
Assessing the accuracy of the surface data obtained from 3D Slicer is beyond the scope of this study, and due to the lack of a gold standard dataset, it is not possible to quantitatively evaluate its precision. However, by refining the extracted surface data within the 3D space using the original images as a reference, we believe that it is possible to obtain even more precise data compared to the automatically extracted models.
(p.11, line196)

**3. The number of samples and diversity (age, gender, body shape, etc.) of the CT data used are not clearly stated. These factors are of particular relevance given their impact on the generalisability of results.**

**Response to Reviewer #1**
Since patient body size data is not publicly available in TCIA, we were unable to obtain it. However, as shown in S1 Fig, we believe that this system has been demonstrated to function effectively regardless of body size, disease type, original image resolution, or dataset size.

**4. Specific solutions to AR issuesThe issue of spatial consistency in AR is mentioned, but no specific approach to solving this problem or future research policy is presented.**

**Response to Reviewer #1**
The resolution of issues related to AR simulation training is not the focus of this study. Therefore, we intend to mention AR only as one of the potential applications of this system.
(p.15, line278)
(p.18, line356)

**5. Verification of cost-effectiveness and comparison with other existing methodsThe assertion that the system is "cost-effective" is not supported by specific evidence or comparative data.**

**A comparative analysis between the proposed system and other extant surgical training methods is absent. This would allow for a more robust demonstration of the system's superiority.
In addition, the long-term effectiveness of the system merits further investigation. The present paper does not examine the long-term effects of training using this system or its impact on actual surgical outcomes.**

**Response to Reviewer #1**
As you pointed out, it is inappropriate to describe this as a cost-effective system since a direct comparison with existing simulation systems is not possible. However, this system is unique in that it allows surface data extracted from a DICOM viewer to be further processed within CAD software, which has not been achieved before.
The construction of simulation systems typically requires significant costs and time for data processing and subsequent simulator design. This system enables healthcare professionals to complete these steps using only free software, potentially reducing costs. Therefore, we will limit our discussion to the possibility of cost reduction.
(p.18, line362)

**Response to Reviewer #2**

**Reviewer #2: General Comments

Thank you for the opportunity to review “Development of Anatomically Accurate Digital Organ Models for Surgical Simulation and Training – A Methodology for Reconstructing and Applying 3D Organ Datasets in Real Space”. Overall, I think this is a worthwhile manuscript. I have a number of edits, suggestions, and comments that I would like the authors to consider before this manuscript is ready for publication.**

**Response to Reviewer #2**
Thank you for reviewing our manuscript and for providing valuable comments that have helped improve it.
Based on the feedback received, we revised the content to remove excessive evaluation and reorganized the discussion. The main achievement of this study is the development of a system that imports organ surface data, obtained through deep learning-based automatic extraction techniques such as 3D Slicer, into CAD software. This allows for modifications based on the original image data to create a more ideal representation.
The data generated by this system is expected to be applicable to various surgical simulations, such as surgical planning and training. However, since no evaluation of the simulator has been conducted, we limit our discussion to the potential applications.
Below, we provide our revisions and responses to the comments received.

**The main comment I have for the authors about this manuscript is that while the guide that the authors created is certainly worthwhile and provides readers with a low-cost mechanism for segmentation and CAD-based design for anatomical structures, the authors overstate how easily the models created through this process could be 3D printed for surgical simulation (not to mention implementation in AR, with no mention of VR). Anatomically accurate simulation not only has to have organs and tissues that are correct size and location, but also, the texture, density, tensile strength (among many other qualities) has to be 1:1 to human tissue. Additive manufacturing practices to print these organs is evolving, and in addition, identifying the correct materials to replicate the different qualities of individual tissue types is still largely unknown (outside of a small number of tissues).**

**Response to Reviewer #2**
As you pointed out, we understand that the manuscript contains expressions that may overstate the ease with which a surgical simulator can be created using this system, potentially leading to misunderstandings.
The primary aim of this study is to establish a workflow using only open-source free software to extract organ surface datasets from DICOM data via 3D Slicer, reconstruct them in Blender while preserving their spatial coordinates, and enable modifications based on the original image data (converted to JPEG format MPR images). This approach allows for manual correction and the addition of organs that cannot be automatically extracted. By semi-automating this process using Python programming, we have significantly reduced the time required to set up this environment.
To validate the usefulness of this protocol, we obtained data from 12 different projects of The Cancer Genome Atlas (TCGA) within the TCIA dataset, each representing a different type of cancer. We measured the processing time for each step and included the data in S2 File and S1 Fig in the manuscript.
The total data processing time required to set up the simulation environment in Blender from DICOM data (corresponding to Fig. 3C in the manuscript) was approximately 7 minutes on average when using 3D Slicer’s Fast mode (which produces a rougher surface model) and 22 minutes when using the Full-resolution mode (which generates more detailed data). Even when including program startup and parameter configuration time, the entire process can be completed within 15 to 30 minutes.
While this organ surface dataset can be adapted for various types of simulators, its use requires additional data processing knowledge and technical skills, meaning that surgeons may not be able to use it immediately without further training. Therefore, in this manuscript, we limit our discussion to the potential applications of the data rather than suggesting its immediate usability in surgical training.
(p.11, line196)
Furthermore, as you pointed out, the success of a simulation depends not only on the system itself but also on the qualitative properties of the organ models. We have been exploring various approaches to address this issue. One of our efforts involves using this system to position porcine organs in anatomically correct human locations to create a surgical simulator. This approach has already been implemented in practice (though not yet published as a manuscript). As an example of potential applications, we present a laparoscopic cholecystectomy training simulator using a porcine liver model.
(p.14, line259)

**I would like the authors to update their manuscript to focus much more on the step-by-step process of their segmentation standard operating procedure to better aid readers in replicating this process simply by reading this manuscript.**

**Response to Reviewer #2**
We have published the overall workflow of the protocol on protocols.io, while the Python scripts required for its implementation have been uploaded to a GitHub repository making them easily accessible via a link from protocols.io.
Additionally, to enable readers to replicate this protocol using specific TCIA data, we have provided a detailed demonstration on GitHub. This demonstration covers the entire process—from obtaining data from TCIA to setting up the simulation environment in Blender (corresponding to Fig. 3C in the manuscript)—including parameter settings and other necessary configurations.
https://dx.doi.org/10.17504/protocols.io.bp2l6d19dvqe/v4
https://github.com/tk1971-Jpn/Demonstration-of-Organ-data-set-creation

**In addition, the authors should only mention 3D-printing or AR as mediums that could then utilize these 3D digital models with the appropriate 3D-printers, software, and materials, and dedicate software developers to incorporate the 3D models into surgical training AR (or VR) software.**

**Response to Reviewer #2**
As mentioned above, this manuscript will only discuss the potential applications of the system.

**Please note, all page numbers listed below are in reference to the PDF proof of the manuscript.

Introduction**

**This section was succinct but provided an OK background to the problem. I would like to see the authors provide an expanded background into the tangible methods of creating surgical simulation models. The vast majority of surgeons would not be able to use a 3D digitized model of an organ(s) for surgical simulation. A thorough discussion of 3D-printing and mixed reality in the world of surgery simulation would enhance this section greatly.**

**Response to Reviewer #2**
Thank you for your valuable suggestions. We have revised the Introduction to include a discussion on the current state and challenges of 3D printing and mixed reality in surgical training.
(p.4, line51)

**Page 13 line71 – please define an acronym the first time you use it (STL).**

**Response to Reviewer #2**
Thank you for your feedback. We have made the necessary revisions.

**Materials and Methods

Page 15 Lines 85-88 – Did the authors begin with their DICOM file in Blender and then move it to 3D Slicer? From my experience it would be flipped. I suggest writing this section in the correct chronological order of operations.

Page 15 lines 90-101 and Page 16 lines 108-117: I am confused about the function of the multi-planar reconstruction images that are automated through a python script. Can the authors please elaborate on the function of this reconstruction images and the benefit of creating them through a script. Why is this an important step instead of simply creating the surface model that 3D Slicer can do?**

**Response to Reviewer #2**
As shown in Fig. 1, this protocol generates multi-planar reconstruction (MPR) images converted from DICOM data into JPEG format. Meanwhile, 3D Slicer is used to extract organ surface data and obtain STL format files.

By importing these data into Blender (more precisely, by referencing MPR images stored in an external folder), users can modify the organ surface data based on the original image data and add organs or blood vessels that could not be automatically extracted, thereby creating a more precise organ dataset.
Since DICOM data cannot be directly displayed in Blender, we first convert and save them as JPEG files for reference. The more reference images available, the more detailed the organ modeling can be. Therefore, we use a Python script to generate the maximum possible number of images at once. Additionally, the script helps manage file names and directory structures, making it easier to load the images into Blender.
(p.10, line170)

**AR Section – Did the authors create any type of UI or interactions for the models beyond the baseline that Apple provides with the AR Kit? Please elaborate if so.

Response to Reviewer #2**
AR is still in the early stages of development, and we have only confirmed that data created with existing software can be applied. Therefore, in this manuscript, we will limit our discussion to mentioning it as one of the potential applications.
(p.13, line239)

**Results**

**Please refer to main comment at the beginning of this review with request to update the results section to largely remove the 3D printed simulator outside of potentially showing how the authors produced their model. This is important, because while the authors 3D printed a surgical simulator based on the segmented model, there appears to be no assessment of the 3D printed simulator.**

**Response to Reviewer #2**
As you pointed out, there is no evaluation of the training effectiveness of the surgical simulator we created, and this is not the focus of this study. Therefore, we will limit our discussion to mentioning it as one of the potential applications of the high-precision digital organ dataset generated by this system.
(p.13, line239)

**Discussion**

**Page 23 lines 252-254: I would like the authors to elaborate more on the creation of organs or tissues that are not auto extracted and how they could create them by simply referring to the image data.**

**Response to Reviewer #2**
In Blender, users can create structures by connecting points or shaping spheres, like sculpting with clay, to form organ shapes. Since Blender allows CT images to be displayed in three orthogonal planes while preserving their original spatial coordinates, users can dynamically adjust the images while connecting points to create blood vessels or shape spheres to match organ boundaries, thereby constructing organ models.
We have added a description of this process to the Discussion section.
